# Extraction/Preconcentration Procedures for Determination of Metal and Organometallic Species in Environmental, Biological, and Food Samples

**DOI:** 10.1155/2019/9465432

**Published:** 2019-01-01

**Authors:** Marcela Zanetti Corazza, Vanessa Egea dos Anjos, Seyyed E. Moradi

**Affiliations:** ^1^Faculty of Science and Technology, Federal University of Grande Dourados, Brazil; ^2^Chemistry Department, State University of Ponta Grossa, Brazil; ^3^Pharmaceutical Sciences Research Center, Faculty of Pharmacy, Mazandaran University of Medical Sciences, Sari, Iran

The development of analytical methods for the determination of trace metals and some organometallic species from complex matrices employing different sample preparation procedures, such as extraction/preconcentration, as well as alternative adsorbent materials, yet is the major challenge for researchers. In addition, the chemical speciation is currently a very important form to enlarge the perspective for environmental, biological, food, and medicinal applications, since toxicity, bioavailability, mobility, and other critical properties of the trace elements depend on this different chemical forms and species in the sample medium. In this special issue on analytical methods of extraction/preconcentration, 6 papers reported different sample preparation for several metals from simple to complex matrix such as geological samples.

One paper published in this special issue reports the application of a novel nanocomposite of nanoporous gold nanoparticles (np-AuNPs) functionalized with 2,2,6,6-tetramethyl-1-piperidinyloxy radical (TEMPO) by the bridge of carboxylate-zirconium-carboxylate chemistry as sensor for the electrochemical detection of H_2_O_2_ at a trace level. In this work, besides demonstrating a strategy for producing large specific surface area nanoporous gold nanoparticles and TEMPO-functionalized np-AuNPs nanocomposite, the electro-oxidation of H_2_O_2_ with a high density of radicals on the TEMPO-np-AuNPs surface is also investigated. The new probe constructed by combination of 4-Carboxy-TEMPO and np-AuNPs shows a double effective enhancement in the electrochemical detection of H_2_O_2_ and wide linear range for H_2_O_2_ detection due to the enzyme-like activity. The sensor had low detection limit, high sensitivity, low cost, anti-interference, good reproducibility, and stability of this nanocomposite with TEMPO-based ligand, contributing to improve the detection of current signal of H_2_O_2_.

Another paper published in this special issue reports the development of a simple, effective, environment-friendly and sensitive analytical method for trivalent antimony determination employing single drop microextraction (SDMM) in water samples. In this work [C4mim][PF6] was employed as the extraction solvent and BPHA was used as the complexing agent making it an analytical method of low cost, simple device, easy operation, and high enrichment efficiency and using low amounts of organic solvent. The use of ionic liquid has been explored in SDME since it does not have detectable vapor pressure, can extract metal ions at room temperature, and avoid several safety problems. So, the introduction of BPHA-[C4mim][PF6] system was not only ecofriendly compared with traditional organic solution, but also efficient for extraction.

In addition, exploring the synthesis and application of materials with ability to preconcentrate metals as well as identify and distinguish different molecules, the paper “Host–Guest Extraction of Heavy Metal Ions with p-t-butylcalix[8]arene from Ammonia or Amine Solutions” reports the behavior and ability of calixarenes (macrocyclic phenolic oligomers) as specific receptor in the extraction of metal ions and/or molecules by modification of the hydroxyl functional group and/or by creating a new cavity size. In calixarenes, the cavity size, position and type of donor groups, and molecular flexibility lead to their high potential for the complexation and extraction of metal ions. It should be noted that the extraction of metal demonstrated to be much higher in some metals when ammonia was used as the aqueous phase.

The fourth paper reported in this special issue “Selective Preconcentration of Gold from Ore Samples” brings the theme of the development of an analytical methodology employing SPE for determination/preconcentration of gold in ores samples. This paper reports the use of an adsorbent Amberlite XAD-16 and N, N-diethyl-N′-benzoylthiourea (DEBT), a selective chelating agent that allowed the selective preconcentration of gold ions from ore samples. It is important to emphasize that low abundance and heterogeneous distribution of gold ions in ore samples require accurate and reliable analytical procedures for its determination. Thus, the accuracy of the proposed method was evaluated by the analyses of two geological samples: Cu-ore and Gold ore (MA-1b) as a certified reference material and the results demonstrated good agreement with the given values. Although there are more sensitive methods applied to similar samples, the proposed method allowed a preconcentration selective of gold ions without any matrix interference using an instrumental technique simple and of low cost (FAAS) when compared to techniques as ICP-MS and GF AAS.

The speciation of Ni in environmental samples has been explored for several years due to carcinogenic potential of water-soluble nickel compounds and nickel tetracarbonyl. However, the analytical techniques for its determination are not always available or easy to use since their limits are threshold values relative to the single species. More specifically, the speciation of Nickel in workplaces airborne particulate is the most important for the assessment of the respiratory health risks. The paper “Development of a New Sequential Extraction Procedure of Nickel Species on Workplace Airborne Particulate Matter: Assessing the Occupational Exposure to Carcinogenic Metal Species” reports a speciation analysis of inorganic Ni compounds such as soluble, sulfidic, metallic, and oxide fractions in airborne particulate matter using a simple selective sequential extraction as sample preparation and analysis by Atomic Absorption Spectroscopy without long evaporation phases. Among the good results obtained by the proposed method stand out the small volumes of solutions and the absence of long evaporation phases. And the last paper published in this special issue, “Simultaneous Determination of Cr, As, Se, and Other Trace Metal Elements in Seawater by ICP-MS with Hybrid Simultaneous Preconcentration Combining Iron Hydroxide Coprecipitation and Solid Phase Extraction Using Chelating Resin”, highlights the benefits of the hybrid preconcentration combing iron hydroxide coprecipitation and solid phase extraction using chelating resin for determination of the oxo-anion forming elements such as Cr, As, and Se and other trace metal elements (Ti, V, Co, Ni, Cu, Zn, Zr, Ge, Cd, Sb, Sn, W, Pb, and U) in seawater, whose standard and guideline values were established in environmental quality standards for water pollution in Japan. In addition, the hybrid preconcentration besides demonstrated recoveries of oxo-anion-forming elements higher than single solid phase extraction using chelating resin (InertSep ME2,60-70 *µ*m in diameter, GL Science Inc., Tokyo, Japan) allowed an extraction in a single pH adjustment (pH 6.0) due to groups functional in the resin surface, such as iminodiacetic acid (p*K*a=2.98) and dimethylamino (p*K*a=10.77) groups.

